# The American System of Practical Medicine

**Published:** 1884-12

**Authors:** 


**The American System of Practical Medicine.** By WILLIAM PEPPER, M.D., LL.D., Provost and Professor of the Theory and Practice of Medicine and of Clinical Medicine in the University of Pennsylvania, assisted by LOUIS STARR, M. D., Instructor of Diseases of Children at the University of Pennsylvania. In five imperial octavo volumes, containing about 1,000 pages each, with illustrations. Volume I. ready February 1, 1885; the other volumes will follow at intervals of about four months. Price per volume, cloth, $5.00; leather, $6.00; half Russia, $7.00.

No physician in America is more thoroughly qualified for the responsible editorship of such a work than Prof. Pepper, and we are confident that the work will be one of which the profession in America will be justly proud. The first volume will appear about February 1, 1885, and the succeeding volumes rapidly thereafter. We predict that the work will immediately take rank above any of the foreign encyclopædias or systems of medicine. The publishers may well feel proud in announcing this magnificent work. For three years it has been in active preparation and it is now in a sufficient state of forwardness to justify them in calling the attention of the profession to it as the work in which, for the first time, American medicine will be thoroughly represented by its worthiest teachers, and presented in the full development of the practical utility which is its distinguishing characteristic. A reference to the published list of contributors, shows the generous rivalry with which the most distinguished men—from the East and the West, from the North and the South, from all the prominent centres of education, and from all the hospitals which afford special opportunities of study and practice—have united in bringing together their vast specialized experience.

